# Rab11 Is Required for Epithelial Cell Viability, Terminal Differentiation, and Suppression of Tumor-Like Growth in the *Drosophila* Egg Chamber

**DOI:** 10.1371/journal.pone.0020180

**Published:** 2011-05-23

**Authors:** Jiang Xu, Lan Lan, Nicholas Bogard, Cristin Mattione, Robert S. Cohen

**Affiliations:** Department of Molecular Biosciences, University of Kansas, Lawrence, Kansas, United States of America; National Institutes of Health (NIH), United States of America

## Abstract

**Background:**

The *Drosophila* egg chamber provides an excellent system in which to study the specification and differentiation of epithelial cell fates because all of the steps, starting with the division of the corresponding stem cells, called follicle stem cells, have been well described and occur many times over in a single ovary.

**Methodology/Principal Findings:**

Here we investigate the role of the small *Rab11* GTPase in follicle stem cells (FSCs) and in their differentiating daughters, which include main body epithelial cells, stalk cells and polar cells. We show that *rab11-null* FSCs maintain their ability to self renew, even though previous studies have shown that FSC self renewal is dependent on maintenance of E-cadherin-based intercellular junctions, which in many cell types, including *Drosophila* germline stem cells, requires Rab11. We also show that *rab11-null* FSCs give rise to normal numbers of cells that enter polar, stalk, and epithelial cell differentiation pathways, but that none of the cells complete their differentiation programs and that the epithelial cells undergo premature programmed cell death. Finally we show, through the induction of *rab11-null* clones at later points in the differentiation program, that Rab11 suppresses tumor-like growth of epithelial cells. Thus, *rab11-null* epithelial cells arrest differentiation early, assume an aberrant cell morphology, delaminate from the epithelium, and invade the neighboring germline cyst. These phenotypes are associated with defects in E-cadherin localization and a general loss of cell polarity.

**Conclusions/Significance:**

While previous studies have revealed tumor suppressor or tumor suppressor-like activity for regulators of endocytosis, our study is the first to identify such activity for regulators of endocytic recycling. Our studies also support the recently emerging view that distinct mechanisms regulate junction stability and plasticity in different tissues.

## Introduction

The Drosophila oocyte develops within a highly organized group of cells called the egg chamber. Each egg chamber consists of a cyst of germ cells and a surrounding monolayer epithelium comprised of somatic follicle cells [1]. The cyst originates from a single cell, the cystoblast, which undergoes four asymmetric rounds of division, each with incomplete cytokinesis, to produce a 16-cell cyst, with only one cell destined to differentiate as the oocyte. Each of the remaining cells adopts a nurse cell fate and is responsible for the synthesis of the vast majority of RNAs and proteins that nurture and pattern the future egg and embryo. The follicle epithelial cells are derived from ovarian mesoderm and function critically in a number of germ-soma signaling events that polarize the oocyte and they are additionally responsible for the secretion of the eggshell and other egg coverings. After secretion of these coverings, the epithelial and nurse cells are targeted for programmed cell death (PCD), leaving the mature egg behind, which is passed through the oviduct and fertilized.

**Figure 1:**
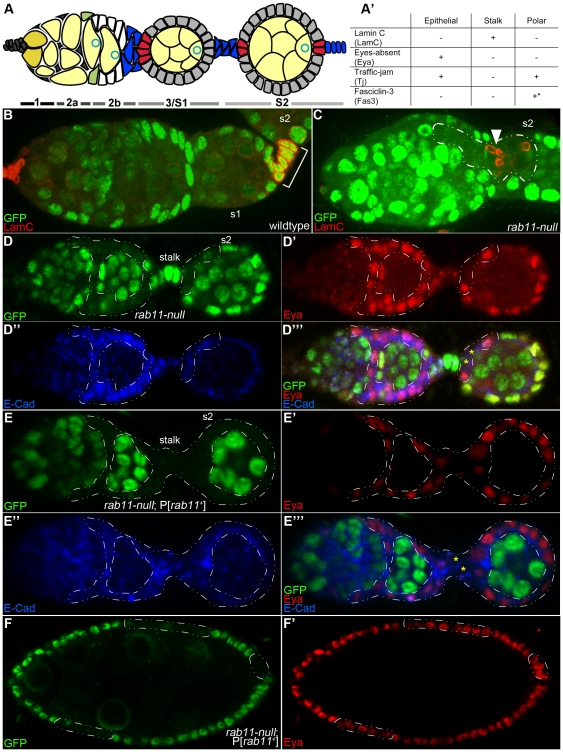
*rab11-null* FSCs give rise to at least two types of cells, one resembling stalk cells and another resembling epithelial cells. (A) Diagram of the Drosophila germarium and a budded stage 2 (s2) egg chamber. Anterior is to the left in this and all subsequent images, unless otherwise noted. Germarial regions 1–3 are indicated below the diagram, where region 3 corresponds to an s1 egg chamber. GSCs (dark yellow) reside at the extreme anterior end of region 1 and give rise to cystoblasts as well as to 2-, 4-, 8- and 16-cell germline cysts (light yellow). Oocyte nucleus, blue circle. Two FSCs (green) reside at the germarial region 2a/2b junction and give rise to undifferentiated pre-follicle cells (white) and three types of differentiated follicle cells: epithelial (grey), polar (red) and stalk (blue). (A′) Expression summary of cell fate markers used in this study, where ‘+’ indicates that the marker is expressed and ‘−’ indicates that the marker is not expressed. The asterisk indicates that Fas3 expression is specific for polar cells, only after stage 3. (B–F′) Confocal images of immunostained germaria and/or egg chambers 10–12 days ACI. (B) Wildtype germarium and s2 egg chamber immunostained for nGFP (green) and LamC (red). Stalk cells are denoted by the bracket. The non-bracketed LamC-positive cells at the left of the figure are niche cap cells, which also over-express LamC [42]. (C) Mosaic germarium, and s2 egg chamber immunostained with nGFP (green) and LamC (red). A *rab11-null* clone is outlined with the dashed white line. The arrowhead points to a cluster of stalk (LamC-plus) cells within the clone. (D-D′″) Mosaic germaria with adjacent s2 egg chambers immunostained for nGFP (green), Eya (red), and E-cad (blue). *rab11-null* clones are outlined with the dashed line. Most of the cells within the clone have adopted an epithelial cell fate as evident by their expression of Eya. The yellow asterisks in the merged image (D′″) highlight a cluster of two, or possibly three, Eya-negative cells, which have presumably adopted a stalk or polar cell fate. (E-E′″) Control *rab11-null*; P[*rab11+*] clones in a germarium and s2 egg chamber immunostained and labeled as in (D-D′″). It may be noted that the germarium shown contains only marked (GFP-negative) follicle cells, indicative of a rare event in which both FSCs were targeted for FLP-induced recombination. (F, F′) Control *rab11-null*; P[*rab11+*] clones in an s8 egg chamber immunostained for nGFP (green) and Eya (red). Marked clones are outlined with the dashed line.

Egg chambers are formed and mature in assembly-line fashion along the anteroposterior axis of tube-like structures called ovarioles. Each of the ∼15 ovarioles that comprise the Drosophila ovary contains an anterior compartment, called the germarium, where egg chambers are assembled from the differentiating progeny of germline and somatic follicle stem cells (GSCs and FSCs, respectively), and a posterior compartment, called the vitellarium where egg chambers mature through 13 morphologically distinct stages (s2–14). The germarium is further subdivided into 4 regions denoted from anterior to posterior as regions 1, 2a, 2b, and 3 (Fig. 1A). The GSCs are located at the anterior tip of germarial region 1, while the FSCs are located at the junction of germarial regions 2a and 2b. Each stem cell population is anchored in place by adherens junctions (Ajs) to neighboring niche cells [2]. Egg chamber formation begins when a GSC divides to produce an anterior cell, which retains its Ajs and GSC identity, and a posterior cell, called a cystoblast, which differentiates. As new cystoblasts are formed, older ones are pushed posteriorly as they divide to produce 2-, 4-, 8- and finally 16-cell cysts. As a 16-cell cyst reaches the region 2a/2b junction it is pushed up against a pool of about 32 pre-follicle (undifferentiated) cells, which causes the cyst to flatten across the full diameter of the germarium. Approximately half of the pre-follicle cells in this pool are derived from one FSC, while the remaining pre-follicle cells are derived from the other FSC [3]. Although mixing between the two pre-follicle cell populations is sometimes observed, it is generally the case that one population migrates over and covers one half (anterior or posterior) of the germline cyst, while the other population migrates over and covers the other half [3]. The cyst and associated pre-follicle cells round up as they move into region 3, where they are known as a stage 1 (s1) egg chamber. Specialized “stalk” cells (see below) at the anterior end of the egg chamber subsequently adopt a wedge-like shape and intercalate to form a single-cell wide bridge that causes the egg chamber to bud into the vitellarium while remaining connected to the next (younger) egg chamber (Fig. 1A).

The faithful assembly and polarization of the egg chamber requires the sequential specification of three distinct follicle cell types through multiple cell-to-cell signaling events. The three follicle cell fates are: anterior and posterior polar cells, which function as signaling centers; stalk cells, which form the above mentioned bridges between adjacent egg chambers and are also responsible for orienting the oocyte within the follicle cell epithelium; and main-body epithelial cells, which secrete the egg shell and regulatory factors that polarize the mature egg and future embryo [1]. Anterior polar cells are specified first, when the Notch signal, Delta, is released from the germline cyst of the region 3 (s1) egg chamber (Fig. 1A). This signal induces 4–6 pre-follicle cells at the junction of germarial regions 2b and 3 to adopt a polar cell fate (Fig. 1A, red cells) [4], [5]. The newly induced polar cells then themselves release Notch and JAK/STAT signals, which act together to induce ∼6 neighboring pre-follicle cells to adopt the stalk cell fate (Fig. 1A, blue cells) [5], [6]. The newly induced stalk cells up-regulate E-cadherin (E-cad), as does the presumptive oocyte of the adjacent, region 2b germline cyst, which eventually results in stable positioning of the oocyte at the posterior end of the cyst (Fig. 1A) [7], [8]. Non-induced pre-follicle cells (that cover more lateral regions of the germline cyst) differentiate as epithelial cells and surround and accompany the cyst as it moves into region 3 as a stage 1 egg chamber (Fig. 1A, grey cells). The region 3 germline cyst then releases Delta, and the whole process of follicle cell specification and oocyte positioning is repeated in the adjacent younger cyst.

As the s1 egg chamber buds into the vitellarium, all but two of the anterior polar cells are targeted for PCD [9] (and see Fig. 1A). Posterior polar cells are also specified at this time, presumably via the conversion of posterior stalk cells to the polar cell fate through continued exposure to the Delta [10]. While polar and stalk cells do not divide after their specification, epithelial cells continue to divide through ∼s6, increasing their cell number from about 30 to over 1000, which is necessary to maintain coverage of the germline cyst, which grows continuously over the course of oogenesis [1].

Although much has been learned about the signaling events that control egg chamber formation and maturation, little is known about the membrane trafficking pathways that underlie these events. We have previously shown that Rab11, best known for its role in trafficking vesicles from recycling endosomes to the plasma membrane, is required in the germline to maintain GSC identity and to correctly orient the cyst within the surrounding epithelium [11]. Both of these requirements are met, at least in part, through Rab11's ability to traffic E-cad from the fusome, a germline specific organelle, to the plasma membrane, and thus, to fortify important contacts between germline and neighboring somatic cells [11]. Given the strong expression of Rab11 in the somatic components of the ovary and the known role of E-cad in maintaining FSC identity and in polarizing epithelial cells [12], [13], we decided to investigate the role of Rab11 in the follicle cell lineage. Unexpectedly, we find that Rab11 is not required for the maintenance of FSC identity. Antibody stains for specific follicle cell fates indicate that *rab11-null* cells are able to respond to inductive signals and initiate specific differentiation programs, but are unable to complete them and are targeted for cell death or are otherwise unable to carry out their intended functions. Finally, we show that the loss of Rab11from cells that have already committed to the epithelial pathway causes them to early arrest early in differentiation, lose cell polarity, and invade the neighboring germline cyst.

## Methods

### Drosophila genetics

Fly culture and crosses were carried out according to standard procedures [14]. The wildtype stock was *w*, *w*+*His2AV::GFP* , or *w+Hrb98DE::GFP* [11], [15]. The *rab11-null*, rab11^Δ*FRT*^, has been previously described [11]. To generate homozygous *rab11*-null clones, we crossed *w; rab11-null/TM3*, *Sb* females to *y w hsp::FLP;* *FRT5377*, *w*+*His2AV::GFP* /*Tm3*, *Sb* or *y w hsp::FLP*; *FRT5377*, *Hrb98DE::GFP* males, where *FRT5337* corresponds to the centromere-proximal FRT insertion element that was used to make the *rab11-null* allele [11]. F1 3^rd^ instar larvae or 2- to 3-day adults were heat shocked for 1 hour at 37°C on 2 consecutive days. Cells (in Sb+ adults) homozygous for the *rab11-null* allele were identified by the absence of GFP fluorescence. Two *sec15*-null mutants were used, *sec15^1^* and *sec15^2^* [16], and identical results were obtained with each. To generate *sec15*-null clones, we crossed *w; FRT82B sec15-null*/*TM3*, *Sb* females to *y w hsp::FLP;* *FRT82B*, *His2AV::GFP/TM3*, *Sb* males. Two- to three-day, Sb+ F1 adults were then heat shocked as described for the *rab11-*null clones.

### Immunocytochemistry and confocal microscopy

Ovaries were fixed and immunostained as described [11], [17]. Primary antibodies were used at the following concentrations: Rat anti-Rab11 (1∶500) [17]; Sec15 (1∶2000; a gift from H. Bellen), Nuf (1∶200) [18], phospho-histone H3 (1∶250; Upstate Biotech.), and GFP (1∶250; Invitrogen). All other primary antibodies were obtained from the Hybridoma bank and used at the following concentrations: E-cad (1∶40), Eya (1∶250), Fas3 (1∶50); Orb (6H4) (1∶20); Fas2 (1∶50); Discs Large (1∶250), ß-integrin (1∶2), and LamC (1∶50). Secondary antibodies were purchased from Jackson labs and used at the manufacturer's recommended concentrations. Apoptotic cells were identified by incubating fixed cells with PhiPhiLux G2D2 (Cal Biochem), which stains activated caspase 3, according to manufacturer's recommended conditions. Stained ovaries were mounted in 4% n-propyl gallate (Sigma) in 90% glycerol, 10% phosphate buffered saline. Images were collected on Olympus 3L Spinning disc or Zeiss Meta 510 laser scanning confocal microscopes.

## Results and Discussion

### Rab11 is not required to maintain FSC identity

Previous studies using partial loss-of-function alleles revealed roles for Rab11 in the germline, but failed to reveal any requirement for the protein in somatic follicle cells [17], [19]. The strong expression of Rab11 and its effectors in somatic follicle cells [[17]; and see below] led us to re-examine Rab11's role in follicle cells using the recently described *rab11-null* allele, rab11^Δ*FRT*^ [11]. We first set out to determine if Rab11 is required in FSCs to maintain stem cell identity. We thought this was a likely possibility given previous findings that FSCs are attached to niche intergermarial cells (IGCs) via E-cad- mediated Ajs [2], [12] and that the delivery of E-cad to the plasma membrane requires Rab11 in a number of different cell types [20], including Drosophila GSCs [11]. We used the FRT-FLP system, to generate *rab11-null* clones that were marked by the loss of nuclear GFP (nGFP) (Methods). As a control, we generated similarly marked wildtype *(rab11+)* clones. All clones were induced at a low frequency to ensure that the vast majority of recovered clones (i.e., GFP-negative cells) were derived from a single parent cell. Unless otherwise noted, all clones were examined 10 or more days after clone induction (ACI) to ensure that they were derived from FSCs; clones derived from FSC daughter cells, or other cells in the follicle cell lineage, would be expected to exit the germarium by day 3 and the entire ovariole by day 9 [2]. As anticipated from these conditions, less than 20% of the examined ovarioles contained marked (GFP-negative) clones and such clones were large, accounting for approximately half of the total number of follicle cells in the germarium and/or young (s1–3) egg chambers. The average size of the *rab11-null* clones was virtually identical to that of the *rab11+* control clones. These findings indicate that *rab11-null* FSCs and their immediate descendants are viable and divide at similar rates to their wildtype counterparts.

We calculated the half-life of the marked *rab11-null* and wildtype FSCs by plotting the percentage of germaria that contained marked follicle cell clones as a function of time (11, 16 and 24 days) ACI. To our surprise, we found that the half-life of marked *rab11-null* FSCs was only slightly lower than that of marked wildtype FSCs (17.2–18.7 days versus 19.8–21.2 days; see Table 1). By comparison, depletion of Rab11 or E-cad from GSCs results in an ∼4-fold reduction in GSC half-life [11], [21]. We conclude from these findings that Rab11 is not required for the maintenance of FSC identity, or for FSC viability. These findings suggest that Rab11 is not required for E-cad trafficking in FSCs. However, we could not test this idea directly, since E-cad levels are low in FSCs and not reliably detected by immunostaining. The strong requirement for Rab11 in E-cad trafficking in GSCs and in the maintenance of GSC identity, versus our results in FSCs, nevertheless indicates that FSCs and GSCs rely on different pathways to maintain needed levels of surface E-cad. Further support for this idea comes from the observation that strongest concentrations of intracellular Rab11 and E-cad in GSCs occur in the fusome [11], an organelle that is absent from FSCs [22], [23].

**Table 1:** *rab11-null* FSCs have a wildtype or near wildtype half-life.

	% marked germaria at different days ACI (total number of germaria counted)			
genotype of marked clones	11	16	24	Half-life
*FRT5377*, *rab11-null*	18.1 (204)	14.8 (203)	11.2 (155)	17.2–19.0 days*
*FRT5377*, *rab11+*	18.7 (315)	15.7 (191)	12.1 (298)	19.8–21.2 days*

*half-lives were calculated for each of the two intervals 11–16 days and 16–24 days using the equation half-life = elapsed time (days)×log 2/log [initial percentage/end percentage], which assumes that FSC loss occurs randomly, and thus linearly over time.

### Rab11 is not required to initiate stalk, polar, and follicle epithelial cell differentiation pathways

To determine whether the daughters of *rab11-null* FSCs could respond to external signals and faithfully initiate stalk, polar, and epithelial cell differentiation programs, we immunostained ovaries with antibodies directed against Lamin C (lamC), which specifically stains stalk cells, or Eyes absent (Eya), which specifically stains epithelial cells (Fig. 1A′). We also used antibodies against Traffic jam (Tj), which stains epithelial and polar cells, but not stalk cells, and/or E-cad, which stains all follicle cells (Fig. 1A′). As before, we carried out our analyses 10 or more days ACI to ensure that all of the recovered *rab11-null* (GFP-negative) cells were derived from *rab11-null* FSCs. We also generated control *rab11-null* clones that contained two copies of a wildtype *rab11* transgene. These clones, designated *rab11-null; P[rab11+]*, were also marked by the loss of nGFP.

The immunostain experiments identified two distinct populations of *rab11-null* follicle cells, one that stained positively for lamC (and negatively for Tj) as expected for stalk cells (Figs. 1B, C; and data not shown), and another that stained positively for Eya (and Tj) as expected for epithelial cells (Figs 1D-D′″; and data not shown). Consistent with their putative stalk cell identity, the *rab11-null*, lamC-positive cells were clustered in small groups of about 6 cells/egg chamber and located at or near the junctions of adjacent egg chambers (see arrowheads in Figs. 1C and 3A, A′). Also, in two rare cases (described below) when bona fide *(rab11+ )* stalk cells and *rab11-null* LamC-positive cells are present in the same egg chamber, the two groups of cells aggregated together. The *rab11-null*, Eya-positive cells formed large clones that included lateral regions of the egg chamber (Fig. 1D-D′″), consistent with their putative epithelial identity. Mosaic egg chambers became severely disorganized at later stages of oogenesis, primarily due to cell death (see below). Unfortunately, this early cell death precluded confirmation of *rab11-null* cell identities by immunostaining for proteins that are expressed in terminally differentiated follicle cells. Nevertheless, the LamC and Eya staining patterns clearly identified two distinct populations of *rab11-null* cells. For simplicity, we will refer to the LamC-positive cells as stalk cells and the Eya-positive cells as epithelial cells, with the understanding that the immunostain experiments show the *rab11-null* cells are able to *initiate*, but not necessarily, complete stalk and epithelial cell differentiation programs.

Two observations indicated that *rab11-null* FSCs also gave rise to follicle cells that initiated polar cell differentiation. First, we observed two populations of LamC-negative (i.e., non-stalk) *rab11-null* cells: one that underwent PCD during s1–3, and another that survived at least through s7 (see below). These longer living cells were generally present in pairs and located at or near the anterior or posterior pole of the egg chamber, consistent with a polar cell or polar cell-like fate, but they never took on the tear-drop shape appearance of fully differentiated polar cells. Second, nearly 100% of the mosaic egg chambers contained clusters of *rab11-null* stalk cells at their anterior and/or posterior ends. Since the specification of the stalk cell fate requires signals from polar cells, we argue that polar cells must have existed (at least) at the time these stalk cell clusters were specified. While we cannot rule out the possibility that all of the recovered *rab11-null* stalk cells were induced by wildtype polar cells, this possibility is difficult to reconcile with the high frequency at which the *rab11-null* stalk cell clusters were recovered; lineage tracing experiments show that stalk and neighboring polar cells generally originate from the same FSC [3]. We interpret these finding to mean that *rab11-null* FSCs give rise to cells that *initiate*, but do not complete, polar cell differentiation. Taken together, we conclude that Rab11 is not required for the initiation of polar cell, stalk cell, or epithelial cell differentiation.

### Epithelial cells derived from rab11-null FSCs are targeted for program cell death during stages 1–3 of oogenesis

**Figure 2:**
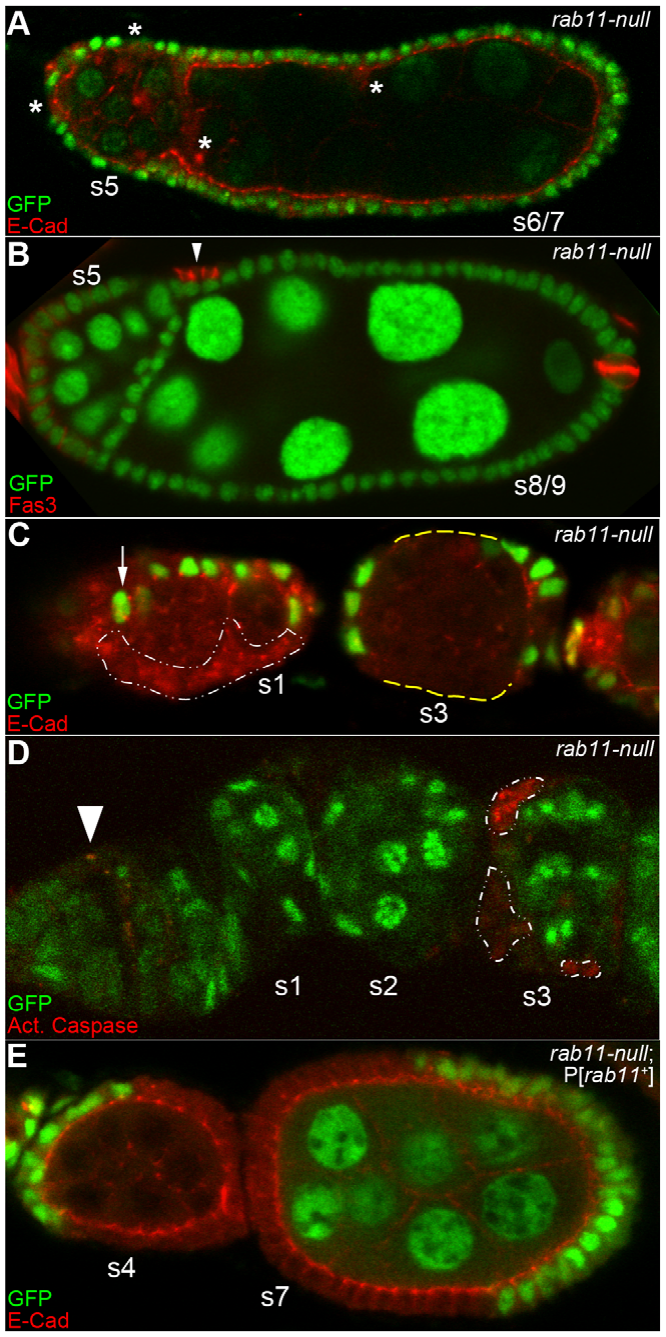
Rab11 is required for the survival of follicle epithelial cells. (A–E) Confocal images of immunostained germaria and/or egg chambers 10–12 days ACI, with *rab11-null* cells marked by the absence of nGFP. All egg chambers are derived from mosaic germaria that contained one *rab11-*null FSC and one wildtype FSC as evidenced by direct examination of the adjacent germarium and/or the presence of *rab11-null* cells in the egg chambers themselves. The stages of the compound and fused egg chambers are estimates based on the size of the nurse cell nuclei, the distance of the fused/compound egg chamber from the germarium, and/or its position relative to other more easily staged egg chambers within the same ovariole. (A) An s5 egg chamber fused to a compound egg chamber containing two ∼s6/7 germline cysts encased in a single continuous epithelium and immunostained for nGFP (green) and E-cad (red). *rab11-null* cells, which are marked with asterisks, are more abundant in the s5 egg chamber than in the adjacent older ones. The positions of the *rab11-null* cells in the compound egg chamber are consistent with a stalk or stalk-like identity (discussed more fully in Text and see Fig. 3). (B) An s5 egg chamber fused to an s8 or s9 egg chamber immunostained for nGFP (Green) and Fas3 (red). The arrowhead points to a cluster of 2–3 *rab11-null* cells that over express Fas-3. While such expression is indicative of a polar cell fate, we cannot rule out a stalk cell fate for these cells as stalk cells that fail to incorporate themselves into a functional stalk are also known to over-express Fas3 [43]. No *rab11-null* epithelial cells are seen in this plane of focus. Other focal planes (not shown) contained no or only a few *rab11-null* epithelial cells (not shown). The two strongly expressing Fas3 cells at the posterior end of the s8/9 egg chamber are wildtype polar cells, although the GFP signal is weak at the focal plane shown. (C) Germarium and s3 egg chamber immunostained for nGFP (Green) and E-cad (red). The arrow points to a putative wildtype FSC. A large *rab11-null* clone in the germarium is outlined in white. The dashed yellow line in the adjacent s3 egg chamber highlights two large gaps in the epithelium as evident by the absence of E-cad expression and also Nomarski imaging (not shown). (D) Germarium and s2 and s3 egg chambers immunostained for nGFP (green) and counterstained for activated caspase 3, a marker for PCD. Approximately half of the *rab11-null* cells (outlined with the dashed line) in the s3 egg chamber stain positively for activated caspase 3. The apparent weaker staining of some of the *rab11-null* cells is not evident at other focal planes (not shown). The activated caspase 3 staining activity at the junction between germarial regions 1 and 2a (arrowhead) is likely to correspond to escort cells, which are known to be targeted for PCD at this stage [44]. (E) Control *rab11-null* ; P[*rab11+*] clones in s4 and s7 egg chambers immunostained for nGFP (green), E-cad (red).

The epithelial cells derived from *rab11-null* FSCs did not persist beyond s4 or s5 (note the absence of GFP-negative follicle cells Figs. 2A, B) and many of the recovered s3–5 egg chambers contained gaps in their epithelial layers (Fig. 2C, yellow dashes). These results suggest that *rab11-null* epithelial cells die during s1–3. The size and frequency of the gaps decreased with egg chamber age and were typically gone by stage 6 (Figs. 2A, B), presumably due to compensatory divisions and replacement by neighboring wildtype cells. To visualize cell death directly, we incubated ovaries 10–12 days ACI with a fluorescently-tagged substrate for activated caspase-3 protein (Methods). Such stains revealed increased cell death of *rab11-null* cells compared to neighboring wildtype cells in s1–3 egg chambers (Fig. 2D). The majority of such deaths occurred in s3 egg chambers, where 50% or more of the *rab11-null* cells stained positively for the death marker (Fig. 2D). By comparison, less than 1% of the wildtype cells ever stained positive for the death marker (Fig. 2D, and data not shown). The premature death of *rab11-null* epithelial cells was rescued by a wildtype *rab11* transgene (Fig. 2E; and see Figs. 1F, F′), and thus, is directly attributable to the loss of *rab11* gene activity.

It is not clear why Rab11 is required for the survival of epithelia cells, but not for the survival of polar, stalk, or FSCs. One possibility relates to the fact that epithelial cells divide much more quickly and/or often than the other cells, and thus, have a greater need for the efficient trafficking of lipids and other membrane materials from intracellular storage compartments (including the RE) to the cell surface [1], [3], [24]. Other hypotheses could also account for the different survival times of *rab11-null* cells, including Rab11-dependent reception and/or recycling of cell type-specific survival signals.

### Stalk cells derived from rab11-null FSCs are viable, but fail to elaborate functional stalks

**Figure 3:**
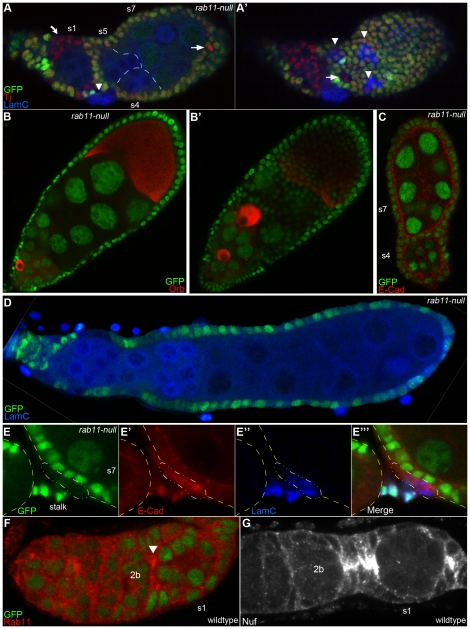
*rab11-null* stalk cells fail to organize themselves into a functional stalk and are associated with fused and compound egg chambers. (A–D) Confocal images of immunostained germaria and/or egg chambers 10–12 days ACI, with *rab11-null* cells marked by the absence of nGFP. (A, A′) Two different focal planes of an s1 (germarial region 3) egg chamber fused to a compound egg chamber containing 3 germline cysts (approximate germline cyst borders outlined with white dashes) immunostained for nGFP (green), LamC (blue), and Traffic jam (Tj) (red). The arrowheads point to putative *rab11-null* stalk (LamC-positive) cell clusters. As described in the text, such clusters contain ∼6 cells each and are located at or near the junctions of fused and compound egg chambers. The arrows point to candidate *rab11-null* polar cells (see Text), while the curved arrow points to a clone of *rab11-null* pre-follicle cells, which also stain positively for Tj. (B, B′) Two different focal planes of a compound egg chamber immunostained for GFP (green) and the oocyte marker, Orb (red). (C) Fused egg chamber immunostained for nGFP (green) and E-cad (red). Anterior at bottom. (D) Massive compound egg chamber immunostained for nGFP (green) and lamC (blue). The LamC-positive nuclei correspond to germ cells and ovariole sheath cells, which are distinguishable from stalk cells by their sizes and position. (E-E′″) Enlarged confocal image of a mosaic stalk cell cluster immunostained for nGFP (green), LamC (blue), and E-cad (red) 5–6 days ACI. The borders of the flanking egg chambers are indicated with the dashed yellow line. The *rab11-null* stalk cells (enclosed in the dashed white line) are excluded from the stalk proper. (F, G) Wildtype germaria immunostained for (F) nGFP (green) and Rab11 (red), or (G) Nuf (white), a Rab11 effector protein [18]. The arrow in (F) points to enriched expression of Rab11 in presumptive stalk and polar cells at the junction of germarial regions 2B and 3 (s1). The region 2b/3 (s1) junction is expanded in (G) as stalk cell formation is more advanced in this particular germarium.

While *rab11-null* FSCs gave rise to cells that initiated stalk cell differentiation (e.g., over-express LamC) none of these cells completed their differentiation program. Specifically, none of the *rab11-null* stalk cells upregulated surface E-cad expression (data not shown) or organized themselves into a recognizable stalk. Consistent with these findings, we recovered many compound egg chambers that contained two or more germline cysts encased in a single continuous epithelium (Figs. 3A, A′). In some cases, a single massive compound egg chamber filled the entire ovariole (Fig. 3D). The compound nature of these egg chambers was confirmed by immunostaining for Orb [25], which revealed at least 2 oocytes in each case (Figs. 3B, B′). Fused egg chambers, i.e., egg chambers that contained a single, stalk-less layer of follicle cells between adjacent germline cysts, were also commonly recovered (Fig. 3C, and see Fig. 2B). All of the examined compound and fused egg chambers contained *rab11-null* stalk (lamC-positive) cells, but the stalk cells were located next to, rather than between, adjacent egg chambers (see arrowheads in Figs. 3A, A′ and Fig. 2B). A similar combination of compound and fused egg chambers are produced by mutants for the Notch and JAK/STAT pathways, which are defective in the specification of polar and stalk cell fates [4], [5], [26], [27].

We wondered whether the lack of fully differentiated *rab11-null* stalk cells reflected a defect in stalk cell differentiation per se or a defect in stalk cell induction, e.g., due to improper specification or differentiation of *rab11-null* polar cells. To distinguish between these possibilities we examined mixed clones that contained both *rab11-null* and wildtype stalk cells. (To this end, we moved the time of our analyses up to 5–8 days ACI to favor recovery of small clones that were induced in pre-follicle cells rather than FSCs.) We recovered two such mixed clones. In each case the wildtype stalk cells were organized into a recognizable stalk (Fig. 3E-E′″), while the neighboring *rab11-null* stalk cells were excluded from the stalk proper (dashed cells in Fig. 3E-E′″). The fact that the two stalk cell populations responded differently to the same polar cell signal (environment) provides strong evidence that Rab11 is required in stalk cells to complete the stalk cell differentiation program and form a stalk. This data further indicates that Rab11 is required cell autonomously in stalk cells for their terminal differentiation. Consistent with this interpretation, we detected very strong expression of both Rab11 and its effector Nuf1 [18] in presumptive stalk and polar cells at the junction of germarial regions 2b and 3 (Fig. 3F, G). The basis for the block in stalk cell differentiation is not clear from our data, but could reflect poor reception or processing of Notch and/or JAK/STAT signals from the neighboring polar cells. Other explanations are also possible, including inefficient trafficking of E-cadherin, which normally accumulates at the leading edge of stalk cells as they intercalate to form a single-cell wide stalk [28].

### Rab11 and its effector, Sec15, are required for the terminal differentiation of epithelial cells

**Figure 4:**
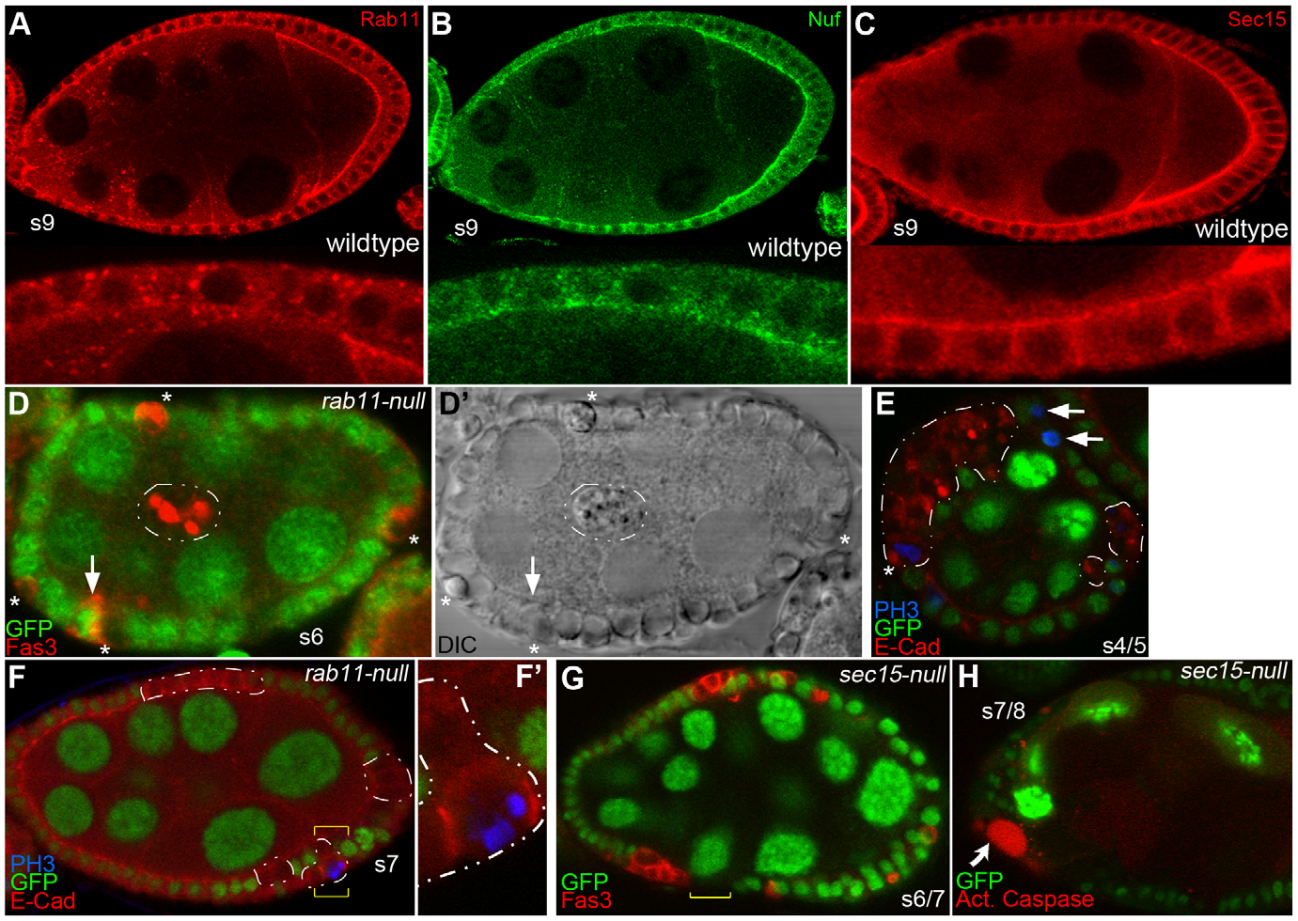
*rab11-null* epithelial cells arrest differentiation early, but continue to divide through s6 or 7, while *sec-15 null* cells arrest differentiation early and are targeted for programmed cell death. (A–C) Confocal images of wildtype s9 egg chambers immunostained for (A) Rab11 (red), (B) Nuf (green), or (C) Sec15 (red). Magnified views are shown at the bottom of each panel. (D) Mosaic s6 egg chamber immunostained for nGFP (green) and Fas3 (red) 4–6 days ACI. The asterisks point to *rab11-null* cells still embedded in the epithelium. A cluster of 3 or 4 *rab11-null* cells that have delaminated from the epithelium and invaded the germline cyst is enclosed in the dashed white line. All of the *rab11-null* cells over-express Fas3 consistent with an early arrest of epithelial cell differentiation (see Text). The arrow points to a wildtype polar cell, which also over-expresses Fas3. (D′) Light micrograph of egg chamber shown in (D). (E) Mosaic s4/5 egg chamber immunostained for nGFP (green), E-cad (red) and phospho-histone 3 (PH3) (blue) 3 days ACI, with *rab11-null* cells enclosed by the dashed white lines. The asterisk marks a dividing (PH3-positive) *rab11-null* cell, while the arrows point to two dividing wildtype cells. (F) Mosaic s7 egg chamber immunostained as in (E), with *rab11-null* cells enclosed by the dashed white lines. (F′) Magnified view of the region bracketed in (F), where a pair of dividing *rab11-null* (PH3-positive) cells are clearly evident. (G) Mosaic *sec15-null* s6/7 egg chamber immunostained for nGFP (green) and Fas3 (red) 2 days ACI. All of the *sec15-null* (GFP-negative) cells over-express Fas3 again consistent with an early arrest of epithelial cell differentiation. The yellow dashed line highlights a gap in the follicle cell epithelium, presumably due to PCD of *sec15-null* cells. (H) Mosaic *sec15-null* s6/7 egg chamber immunostained for nGFP (green) and counterstained for activated caspase-3 two days (red) ACI. The arrow points to an apoptotic *sec15-null* cell.

**Figure 5:**
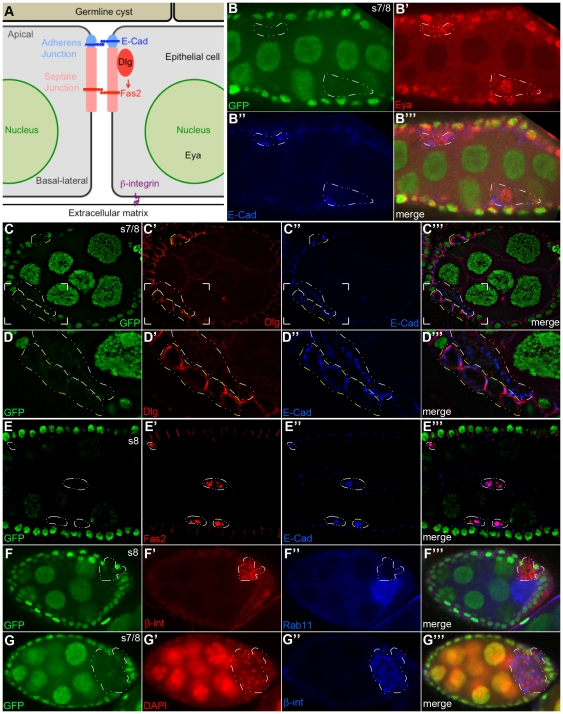
*rab11-null* follicle cells lose their polarity, delaminate from the epithelium and invade the neighboring germline cyst. (A) Schematic diagram of follicle epithelial cell polarity. Markers used in this study are highlighted [adapted from [13]]. (B–G″) Confocal images of mosaic stages 7/8 egg chambers 4–6 days ACI. The *rab11-null* clones are marked by their absence of GFP expression and are outlined with dashed lines. (B-B′″) nGFP (green), Eya (red), E-cad (blue). All of the *rab11-null* cells stain positive for Eya, consistent with an epithelial cell fate. In contrast to the strict apical expression pattern of E-cad in neighboring wildtype cells, the protein is highly enriched in intracellular compartments in the *rab11-null* cells (also see D″ and E″). (C-C′″) nGFP (green), Discs large (Dlg) (red), E-cad (blue). (D-D′″) Enlarged views of the bracketed regions shown in (C-C′″). Note that *rab11-null* cells that are still embedded in the epithelium (outlined in yellow) exhibit wildtype or near wildtype (basolateral) expression patterns for Dlg and mostly normal (mostly apical) expression pattern for E-cad. In contrast, the *rab11-null* cells that have delaminated from the epithelium (outlined in white) exhibit a vesicular staining pattern for E-cad, while Dlg is dispersed throughout the cell and /or completely absent. (E-E′″) nGFP (green), Fas2 (red), E-cad (blue). Three clusters of delaminated *rab11-null* cells are outlined. Each cluster contains two cells. None of the cells exhibit apical-basal polarity as evident by the vesicular-like staining pattern of both Fas2 and E-cad. (F-F′″) nGFP (green), ß-integrin (ß-int) (red), Rab11 (blue). Note, the donut-shape distribution pattern of ß-int, which suggest that the some of the protein is still on the cell surface. All of the other examined cell surface markers exhibit a strictly intracellular staining pattern in delaminated cells. The circled *rab11-null* clone, along with the one shown in (G) is situated in a bubble between the epithelium proper and the germline cyst, which partially accounts for the weak ß-int signal in the flanking wildtype epithelial cells. Nevertheless, the ß-int signal was reproducibly more intense in the *rab11-null* epithelial cells than in wildtype epithelial cells. (G-G′″) nGFP (green), DAPI (red), ß-int (blue). A large (>50 cells) *rab11-null* clone in the posterior portion of the egg chamber is circled. Note that the ß –int staining pattern in this clone is more vesicular in nature than that in the previous panels. Most other similarly large clones were also located in the posterior portion of the egg chamber and like the one shown wedged between the follicle cell epithelium and the oocyte.

Because the epithelial cells derived from *rab11-null* FSCs died shortly after they were specified, the experiments described above could not test whether Rab11 is needed to maintain epithelial cell polarity and/or other aspects of epithelial cell behavior. Such roles seemed likely given that Rab11 and two of its best characterized effectors, Sec15 and Nuf, are expressed in follicle epithelial cells throughout oogenesis (Figs. 4A–C; and data not shown). To investigate a possible role for Rab11 in more mature epithelial cells, we shifted our analysis point up to 2–6 days ACI to favor the recovery of clones induced in pre-follicle and/or young s1–3 epithelial cells. We reasoned that such cells would survive into late stages of oogenesis, provided that Rab11's role in epithelial cell survival is transient in nature. This approach proved useful as nearly half of the recovered s4–8 egg chambers contained clones of *rab11*-*null* follicle cells (Fig. 4). The vast majority of thesecells were located in lateral regions of the egg chamber (Figs. 4D–F) and/or over-expressed Eya (Fig. 5B; and data not shown) consistent with the idea that they had adopted an epithelial cell fate. This was the expected result, as clone induction requires cell division and stalk and polar cells stop dividing by s1. Unlike the epithelial cells recovered from *rab11-null* FSCs, the ones examined 2–6 ACI days remained viable through s8 (Figs. 4, 5). (We could not look later than s8, without delaying our analyses to 6–8 days ACI, which had the complication of recovering egg chambers derived from *rab11-null* FSCs). The examination of clones 2–6 days ACI also allowed us to recover s4–8 epithelial cells homozygous for a null allele of *sec15* (Fig. 4G), which encodes a component of the exocyst and a Rab11 effector required for the docking of vesicles to the plasma membrane [29], [30], [31]. The vast majority of recovered egg chambers contained a completely wildtype germline. Rare egg chambers with a mutant germline cyst were not analyzed to eliminate complications in data interpretation.

All of the recovered s4–8 *rab11-null* epithelial cells arrested differentiation early as evident by their strong expression of Fas3 (Fig. 4D), a protein that is normally strongly expressed only in s3 and younger epithelial cells [32]. Three observations together rule out the alternative possibility that the Fas3-positive *rab11-null* cells trans-differentiated from an epithelial to a polar cell fate, which is also characterized by strong expression of Fas3. First, as mentioned above, all of the *rab11-null* cells strongly expressed Eya (Fig. 5B′), which is characteristic of epithelial cells, but not of polar cells [33] (Fig. 1B′). Second, the vast majority of *rab11-null* cells, like normal epithelial cells, divided through s6, as evident by phospho-histone 3 expression (Fig. 4E–F′), whereas, polar cells do not divide beyond s1. Third, the *rab11-null* cells were not incorporated into border cell clusters and they did not exhibit border cell-type migration. Border cell clusters normally arise at stage 9, when anterior polar cells recruit ∼6 neighboring cells into a migration-competent cell cluster that delaminates from the epithelium and migrates to the nurse cell-oocyte border [34]. The *rab11-null* cells delaminated, but they did so prior to s9 and they did not recruit other (i.e., neighboring wildtype) cells into the cluster. Also, the delaminated *rab11-null* cells migrated in random directions, rather than toward the nurse cell-oocyte border (see below). The *sec15-null* cells also arrested differentiation early as evident by their strong expression of Fas3 (Fig. 4G). However, in contrast to the *rab11-null* cells, which survived for up to 6 days ACI, nearly half of the *sec15-null* cells were targeted for PCD by 2 days ACI (Fig. 4H). While these data are consistent with the idea that Sec15 is an effector of Rab11 in follicle cell differentiation, they further indicate that Sec15 has a Rab11-independent role in cell viability, which is not unexpected given Sec15's well-described role as a Rab8 effector in the docking of Golgi-derived vesicles to the plasma membrane [16], [31].

### Rab11 behaves as a tumor suppressor-like protein in follicle epithelial cells

The *rab11-null* epithelial cells exhibited a variety of neoplastic tumor-like behaviors, including the above mentioned block in differentiation, loss of cell polarity, and the ability to invade neighboring cell masses. The loss of cell polarity was initially apparent in the gross morphology of the mutant cells, which became progressively more rounded over time. By 5 days ACI, a majority of the *rab11-null* cells were completely rounded up and displaced from the epithelium (Fig. 4D′ and Figs. 5B–G). The loss of cell polarity inferred by the rounded up morphology was confirmed by immunostaining for protein markers of cell polarity (Fig. 5A). Most telling, E-cad and Discs large (Dlg), which establish apical-basal membrane polarity through their organization of adherens and septate junctions [28], [35], [36], [37], respectively, were both mis-localized in *rab11-null* cells (Figs. 5C–D′″). In the case of Dgl, mis-localization was restricted to delaminated cells (e.g., see area circled in white in Fig 5D′), where no Dlg was detected, presumably because it was completely dispersed throughout the cytoplasm and/or degraded. In contrast, E-cad was mis-localized in all *rab11-null* cells (Fig. 5), albeit more completely so in the ones that had delaminated, where all of the protein was trapped in intracellular compartments (e.g., see cells circled in white in Fig. 5D″); E-cad was localized to the plasma membrane of non-delaminated cells (Fig. 5D″, cells circled in yellow), although it was no longer enriched apically as it is in wildtype epithelial cells. We interpret these findings to mean that *rab11-null* cells first lose E-cad localization, and delaminate from the epithelium shortly before or after subsequent loss mislocalization and/or loss of Dlg. A loss of cell polarity was also revealed by immunostaining for Fasciclin 2 (Fas2) (Fig. 5E), a putative membrane anchor for Dlg [13] and ß-integrin a basal membrane marker (Figs. 5F, G). Previous studies with Drosophila embryos revealed a role for Rab11 in maintaining Ajs [38], but did not uncover a requirement for Rab11 in maintaining the localization of Dlg, Fas2 and/or other known or putative components of septate junctions. One possible explanation for this difference is that the embryonic studies used dominant negative and hypomorphic alleles of *rab11*, which may not have completely eliminated Rab11 function.

The invasive behavior of the *rab11-null* cells is distinct from that described for mutations in characterized Drosophila tumor suppressor genes (tsgs), which include the septate junction organizers, *discs large*, *scribble*, and *lethal giant larvae*, and two regulators of endocytosis, *avalanche*, and *rab5* [37], [39]. Thus while previously characterized tsg mutant cells invade surrounding tissues as large multi-layered sheets that remain attached to the epithelium [40], the *rab11-null* cells were often completely detached from the epithelium and were in groups containing as few as two cells (e.g., Fig. 5E′″) or as many as 50 or more (e.g., Fig. 5G′″). In this regard, the invasive behavior of *rab11*-null cells more closely parallels the behavior of metastatic tumor cells of higher animals [41]. Nevertheless, we wish to emphasize that we have no direct evidence that *rab11-null* cells actively migrate and in fact cannot rule out the possibility that their “invasion” of the germline cysts occurs in a passive fashion, e.g., by their inability to maintain adhesive contacts with neighboring wildtype epithelium cells and subsequent exclusion from the epithelium.

In contrast to bona fide tumor cells, the vast majority of *rab11-null* epithelial cells stopped dividing on schedule, i.e., at s6 (Figs. 4E, F, and data not shown). A few exceptional cells divided during s7 (Fig. 4F, F′), but none divided after that. It is noteworthy that all of the exceptional (late dividing) cells delaminated from basal side of the epithelium, which may have precluded them from receiving Delta from the germline, which is known to promote a switch from a mitotic cell cycle to an endocycle at s7. The overwhelming majority of *rab11-null* cells delaminated from the apical side of the epithelium (e.g., Figs. 4D, E) and presumably, then, received Delta, which may account for their mitotic arrest. Drosophila's previously characterized tsgs also have no or only subtle roles in suppressing follicle cell over-proliferation [37]. Indeed, the evidence that these genes suppress over-proliferation stem entirely from analyses of larval tissues, most notably imaginal discs. Whether suppression of over-proliferation in larval tissues is fundamentally different, or simply easier to demonstrate, than suppression of over-proliferation in adult follicle epithelial cells is unclear. To date, we have been unable to recover *rab11-null* clones in imaginal discs and other larval tissues, which may reflect a unique role for Rab11 in the survival of such cells. In light of these data, we propose that Rab11 protein be considered as tumor suppressor-*like* protein.
